# Melatonin Protects Band 3 Protein in Human Erythrocytes against H_2_O_2_-Induced Oxidative Stress

**DOI:** 10.3390/molecules24152741

**Published:** 2019-07-28

**Authors:** Rossana Morabito, Alessia Remigante, Angela Marino

**Affiliations:** 1 Department of Chemical, Biological, Pharmaceutical and Environmental Sciences, University of Messina, Viale F. Stagno D’Alcontres 31, 98166 Messina, Italy; 2 Institute of Pharmacology and Toxicology, Paracelsus Medical University, 5020 Salzburg, Austria

**Keywords:** Melatonin, Band 3 protein, SO_4_^=^ uptake, oxidative damage, H_2_O_2_

## Abstract

The beneficial effect of Melatonin (Mel), recognized as an anti-inflammatory and antioxidant compound, has been already proven to prevent oxidative stress-induced damage associated to lipid peroxidation. As previous studies modeled the impact of oxidative stress on Band 3 protein, an anion exchanger that is essential to erythrocytes homeostasis, by applying H_2_O_2_ at not hemolytic concentrations and not producing lipid peroxidation, the aim of the present work was to evaluate the possible antioxidant effect of pharmacological doses of Mel on Band 3 protein anion exchange capability. The experiments have been performed on human erythrocytes exposed to 300 μM H_2_O_2_-induced oxidative stress. To this end, oxidative damage has been verified by monitoring the rate constant for SO_4_^=^ uptake through Band 3 protein. Expression levels of this protein Mel doses lower than 100 µM have also been excluded due to lipid peroxidation, Band 3 protein expression levels, and cell shape alterations, confirming a pro-oxidant action of Mel at certain doses. On the other hand, 100 µM Mel, not provoking lipid peroxidation, restored the rate constant for SO_4_^=^ uptake, Band 3 protein expression levels, and H_2_O_2_-induced cell shape alterations. Such an effect was confirmed by abolishing the endogenous erythrocytes antioxidant system. Therefore, the present findings show the antioxidant power of Mel at pharmacological concentrations in an in vitro model of oxidative stress not associated to lipid peroxidation, thereby confirming Band 3 protein anion exchange capability measurement as a suitable model to prove the beneficial effect of Mel and support the use of this compound in oxidative stress-related diseases affecting Band 3 protein.

## 1. Introduction

The efficiency of Band 3 protein, the anion exchanger present in million copies on erythrocytes membrane and accounting for ion balance, erythrocytes deformability and tissue oxygenation [1], can be monitored by determining the rate constant for SO_4_^=^ uptake, which is slower and more easily detectable than Cl^-^ or HCO_3_^-^ uptake [2,3]. SO_4_^=^ uptake measurement has been previously confirmed as a suitable tool to verify the impact of oxidative conditions on erythrocytes homeostasis [4]. In this regard, H_2_O_2_, used to produce oxidative stress at not hemolytic concentrations [5], impacts on the phospholipid bilayer arrangement and the integral proteins associated to cytoskeleton, including Band 3 protein [6]. Our previous investigations demonstrated a reduction in Band 3 protein anion exchange capability and protein expression levels after a short-term period of exposure (30 min) to 300 µM H_2_O_2_ associated neither to lipid peroxidation, nor to membrane –SH groups oxidation (mostly belonging to Band 3 protein) or MetHb formation [4]. More recently, SO_4_^=^ uptake measurement has been shown as a valid tool to detect oxidative damage in the case of oxidative stress-related diseases, such as systemic sclerodermia, diabetes, and canine leishmaniasis [7,8,9,10]. In addition, the role of antioxidants in preventing oxidative damage at Band 3 levels has also been investigated [11].

Among antioxidants assumed by food or used in clinics to counteract oxidative conditions often associated to pathologies, melatonin (*N*-acetyl-5-methoxy-tryptamine) has been considered in the present study.

Melatonin (Mel) is involved in photoperiodism as a neuroendocrine transducer, synthesized in the pineal gland as central site of release, detected in serum at nM physiological concentrations and locally produced in other tissues such as gastrointestinal tract [12]. Its anti-inflammatory activity, immune enhancement, prevention of carcinogenesis and tumor promotion are already known [13,14], while its antioxidant power remains not completely clarified [15,16], as both antioxidant and pro-oxidant activity are described, depending on concentration and cell target [17,18]. Though plenty of experiments showed that Mel is a powerful free radical scavenger and antioxidant at different extent detected on lipids and proteins [19], other in vitro studies highlight its context specific pro-oxidant actions, namely in cancer cells [14].

On these premises and taking into account that erythrocytes are a unique and suitable cell model to investigate the response to oxidative stress, due to their simple metabolism and sensitivity to oxidation [20], the aim of the present study was to prove a possible antioxidant effect of Mel on an in vitro model of H_2_O_2_-induced oxidative stress on human erythrocytes [4]. The hypothesis is that Mel may exert its antioxidant power on an in vitro model that is not associated to lipid peroxidation. Moreover, Band 3 protein could be a target to prove the antioxidant effect which could be, in turn, adopted against oxidative stress-related diseases affecting Band 3 protein [8,9].

In order to understand whether Mel displays anti- or pro-oxidant action on Band 3 protein, lipid peroxidation possibly induced by Mel has been monitored [21]. To this end, pharmacological Mel doses (µM) have been used according to other in vivo and in vitro studies [15,19]. Successively, the Mel antioxidant effect has been verified by exposing erythrocytes to 300 μM H_2_O_2_ and then evaluating cell shape alterations, rate constant for SO_4_^=^ uptake through Band 3 protein along with Band 3 protein expression levels [22,23]. In a separate experimental set, a specific inhibitor of catalase enzyme, playing a pivotal role in defending erythrocytes at H_2_O_2_ concentrations comprised between 10 and 300 µM [5,22] has been also used to prove the Mel antioxidant effect in the absence of an endogenous antioxidant system.

## 2. Results

### 2.1. Thiobarbituric-Acid-Reactive Substances (TBARS) Levels

**Figure 1:**
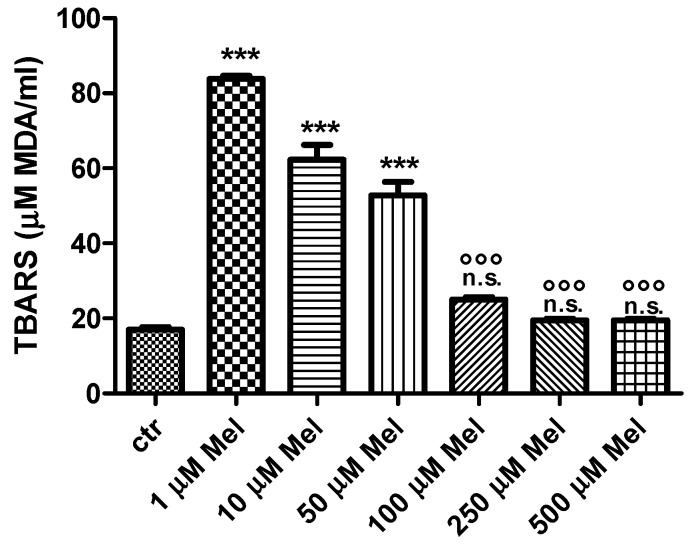
TBARS levels measured as µM malondialdehyde/mL erythrocytes after treatment with Mel at different concentrations. *** *p* < 0.001 vs. ctr; n.s. not significant vs. ctr; ^°°°^ *p* < 0.001 vs. either 1 µM or 10 µM or 50 µM Mel, as determined by one way ANOVA followed by Bonferroni’s *post hoc* test (N = 10).

As shown in Figure 1, thiobarbituric-acid-reactive substances (TBARS) levels after either 1 µM or 10 µM or 50 µM Mel treatment were significantly higher than control (*p* < 0.001). After treatment with either 100 µM or 250 µM or 500 µM Mel, TBARS levels were significantly lower (*p* < 0.001) than those determined after exposure to either 1 µM or 10 µM or 50 µM Mel, while remaining unchanged with respect to control.

### 2.2. Cell Shape

**Figure 2:**
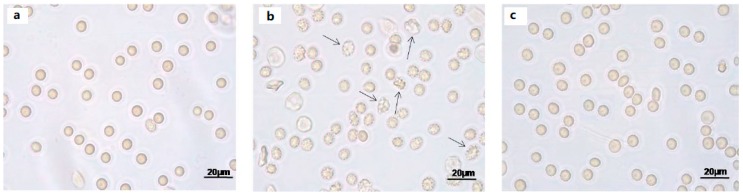
Light microscope observations of untreated (ctr) (**a**), 1 µM Mel- (**b**) and 100 μM Mel-treated erythrocytes (**c**), at 60 min of Mel incubation. Note the significant morphological changes in **b** (arrows), where 97% altered cells were observed.

Light microscope observations clearly show cell shape changes after 1 μM Mel treatment (Figure 2b) that were not observed after treatment with 100 μM Mel (Figure 2c).

### 2.3. SO_4_^=^Uptake Measurement

#### 2.3.1. H_2_O_2_ Treatment

**Figure 3:**
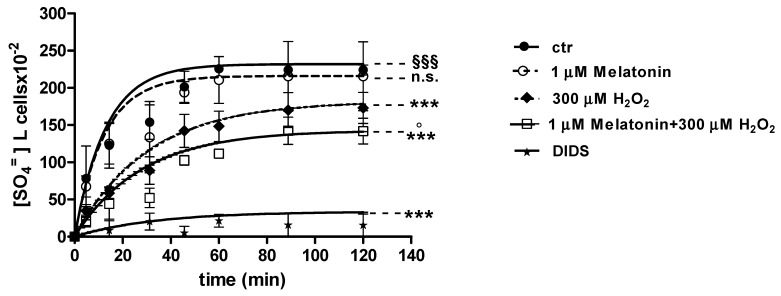
Time course of SO_4_^=^ uptake in untreated erythrocytes (ctr) and erythrocytes treated with 300 µM H_2_O_2_, with or without pre-exposure to 1 µM Mel. n.s. not significant vs. ctr; *** *p* < 0.001 vs. ctr, 1 µM Mel + 300 µM H_2_O_2_^°^ *p* < 0.001 vs. 300 µM H_2_O_2_, ^§§§^ *p* < 0.001, as determined by one way ANOVA followed by Bonferroni’s post hoc test (N = 8).

**Figure 4:**
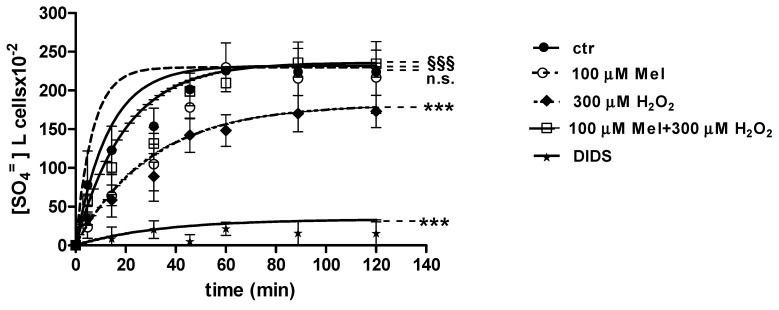
Time course of SO_4_^=^ uptake in untreated erythrocytes (ctr) and erythrocytes treated with 300 µM H_2_O_2_, with or without pre-exposure to 100 µM Mel. n.s. not significant vs. ctr; *** *p* < 0.001 vs. ctr, 100 µM Mel + 300 µM H_2_O_2_^§§§^ *p* <0.001 vs. 300 µM H_2_O_2_, as determined by one way ANOVA followed by Bonferroni’s post hoc test (N = 8).

Curves depicted in Figure 3 and Figure 4 describe SO_4_^=^ uptake in different experimental conditions, as a function of time. The rate constant for SO_4_^=^ uptake, accounting for velocity of this process, progressively increased and reached equilibrium within 45 min, with a value of 0.076 ± 0.001 min^−1^ in control erythrocytes (Table 1). Pre-exposure to either 1 (Figure 3) or 100 µM Mel (Figure 4) did not alter this parameter, as a rate constant of, respectively, 0.080 ± 0.001 min^−1^ and 0.072 ± 0.001 min^−1^ was observed (*p* < 0.001, Table 1). Erythrocytes treated with 300 µM H_2_O_2_ exhibited a rate constant of 0.046 ± 0.001 min^−1^ that was significantly lower than control (*p* < 0.001, Table 1) while being comparable to erythrocytes pre-exposed to 1 µM Mel and then to 300 µM H_2_O_2_ (0.048 ± 0.001 min^−1^). Erythrocytes pre-exposed to 100 µM Mel and then to 300 µM H_2_O_2_ exhibited a rate constant of 0.074 ± 0.001 min^−1^ not significantly different with respect to control, while significantly different with respect to 300 µM H_2_O_2_. Treatment with 10 µM DIDS, which was applied at the beginning of SO_4_^=^ medium incubation, completely blocked SO_4_^=^ uptake (0.017 ± 0.001 min^−1^, *p* < 0.001, Table 1).

The SO_4_^=^ amount trapped by erythrocytes exposed to 1 and 100 µM Mel at 45 min of incubation in SO_4_^=^ medium (192.8 ± 23 and 229.8 ± 14.5, respectively) was not significantly different with respect to control cells (201.3 ± 21), while, after exposure to 300 µM H_2_O_2_ (142.2 ± 22.2 mM), it was significantly lower than control (*p* < 0.001, Table 1). In DIDS-treated cells, the SO_4_^=^ intracellular amount at 45 min of incubation (4.75 ± 9) was significantly lower than both control and experimental conditions (Table 1).

**Figure 5:**
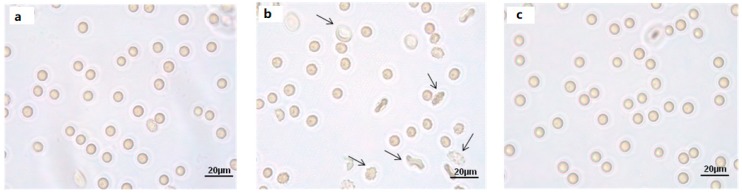
Light microscope observations of untreated (ctr) (**a**), 300 µM H_2_O_2_- (**b**) and 100 μM Mel + 300 µM H_2_O_2_-treated erythrocytes (**c**), at 90 min of SO_4_^=^ medium incubation. Note the significant morphological changes in **b**, where, 70% altered cells were observed.

Light microscope observations at 90 min of incubation in SO_4_^=^ medium show morphological changes in 300 μM H_2_O_2_-treated erythrocytes (Figure 5b), not seen in erythrocytes treated with 100 μM Mel followed by 300 μM H_2_O_2_ (Figure 5c).

#### 2.3.2. H_2_O_2_ and 3-AT Treatment

**Figure 6:**
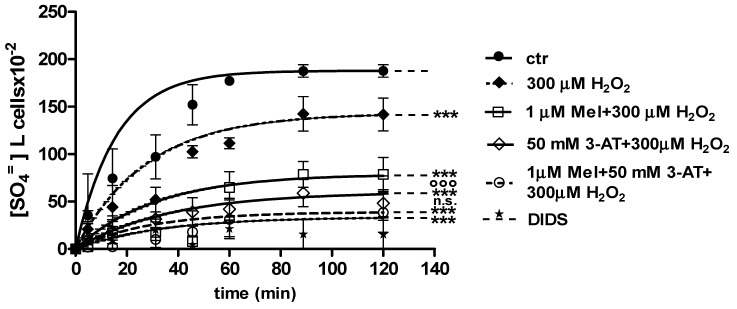
Time course of SO_4_^=^ uptake measured in untreated erythrocytes (ctr) and in erythrocytes treated with 1 µM Mel + 300 µM H_2_O_2_ with or without pre-exposure to 50 mM 3-AT. *** *p* < 0.001 vs. ctr; 50 mM 3-AT + 300 µM H_2_O_2_^°°°^ *p* < 0.001 vs. 300 µM H_2_O_2_; 1 µM Mel + 50 mM 3-AT + 300 µM H_2_O_2_ n.s. not significant vs. 50 mM 3-AT + 300 µM H_2_O_2_ as determined by one way ANOVA followed by Bonferroni’s post hoc test (N = 8).

**Figure 7:**
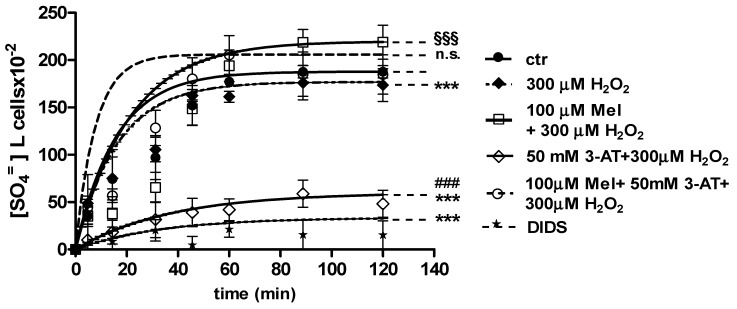
Time course of SO_4_^=^ uptake measured in untreated erythrocytes (ctr) and in erythrocytes treated with 100 µM Mel + 300 µM H_2_O_2_ with or without 50 mM 3-AT. *** *p* < 0.001 vs. ctr; 100 µM Mel + 50 mM 3-AT + 300 µM H_2_O_2_ n.s. not significant vs. ctr, 100 µM Mel + 300 µM H_2_O_2_^§§§^ *p* < 0.001 vs. 300 µM H_2_O_2_; ^###^ *p* < 0.001 vs. 100 µM Mel + 50 mM 3-AT + 300 µM H_2_O_2_ as determined by one way ANOVA followed by Bonferroni’s post hoc test (N = 8).

Figure 6 and Figure 7 show the kinetics of SO_4_^=^ uptake in the case of catalase inhibition. With regard to Figure 6, when 50 mM 3-AT and 1 µM Mel were applied before 300 µM H_2_O_2_, the rate constant for SO_4_^=^ uptake (0.019 ± 0.001 min^−1^) was significantly lower than control (0.076 ± 0.001 min^−1^), while not being significantly different with respect to 50 mM 3-AT + 300 µM H_2_O_2_ treatment (0.018 ± 0.001 min^−1^) (Table 1). The SO_4_^=^ amount trapped by erythrocytes treated with 50 mM 3-AT + 1 µM Mel + 300 µM H_2_O_2_ was 38.4 ± 11 (Table 1).

With regard to Figure 7, when 50 mM 3-AT and 100 µM Mel were applied before 300 µM H_2_O_2_ treatment, the rate constant for SO_4_^=^ uptake (0.077 ± 0.001 min^−1^) was not significantly different with respect to both control (0.076 ± 0.001 min^−1^) and 100 µM Mel + 300 µM H_2_O_2_ treatment (0.074 ± 0.001 min^−1^). This latter value was significantly higher than that one determined after 300 µM H_2_O_2_ treatment (0.046 ± 0.001 min^−1^, Table 1). SO_4_^=^ amount trapped by erythrocytes treated with 50 mM 3-AT + 100 µM Mel + 300 µM H_2_O_2_ was 219 ± 17 (Table 1).

### 2.4. Band 3 Protein Expression Levels Determination

**Figure 8:**
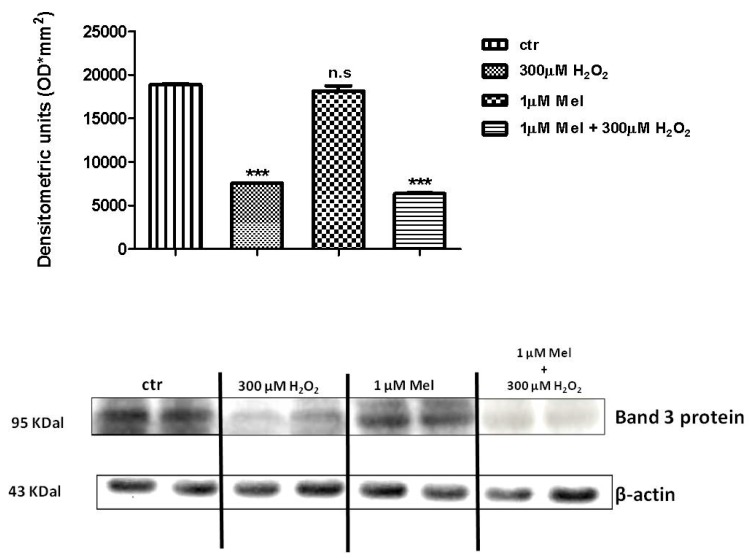
Expression levels of Band 3 protein measured in untreated erythrocytes (ctr), or in 300 µM H_2_O_2_- or in 1 µM Mel or in 1 µM Mel + 300 µM H_2_O_2_-treated erythrocytes, detected by Western blot analysis. *** *p* < 0.001 vs. ctr; n.s. not significant vs. ctr, as determined by one way ANOVA followed by Bonferroni’s *post hoc* test (N = 5).

**Figure 9:**
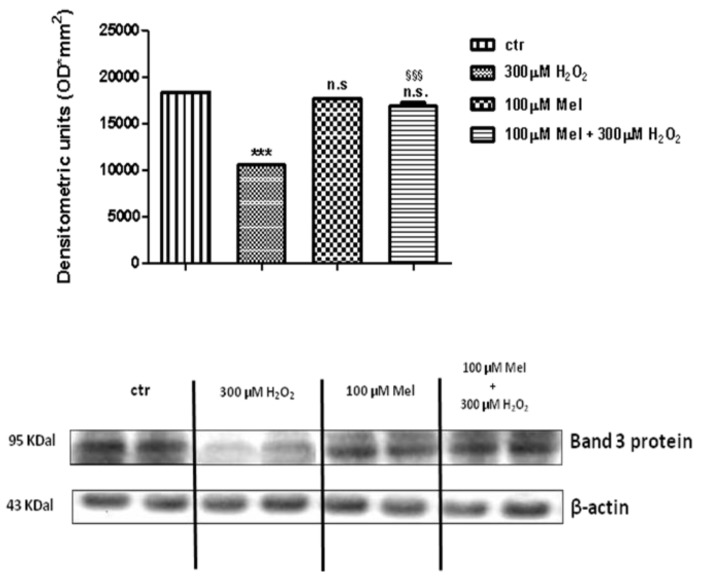
Expression levels of Band 3 protein measured in untreated erythrocytes (ctr), or in 300 µM H_2_O_2_- or in 100 µM Mel or in 100 µM Mel + 300 µM H_2_O_2_-treated erythrocytes, detected by Western blot analysis. *** *p* < 0.001 vs. ctr; n.s. not significant vs. ctr; ^§§§^ *p* < 0.001 vs. 300 µM H_2_O_2_, as determined by one way ANOVA followed by Bonferroni’s *post hoc* test (N = 5).

Figure 8 and Figure 9 show that Band 3 protein levels in either 1 µM Mel- or 100 µM Mel-treated erythrocytes were not significantly different with respect to those determined in untreated erythrocytes (control), while, after treatment with 300 µM H_2_O_2_, they were significantly lower than control (*p* < 0.001). A not significant difference (*p* < 0.001) between expression levels in 1 µM Mel + 300 µM H_2_O_2_-treated erythrocytes and those measured in 300 µM H_2_O_2_-treated erythrocytes was also observed (*p* < 0.001, Figure 8). On the other hand, a significant difference (*p* < 0.001) between expression levels in 100 µM Mel + 300 µM H_2_O_2_-treated erythrocytes and those measured in 300 µM H_2_O_2_-treated erythrocytes was observed (*p* < 0.001, Figure 9).

**Figure 10:**
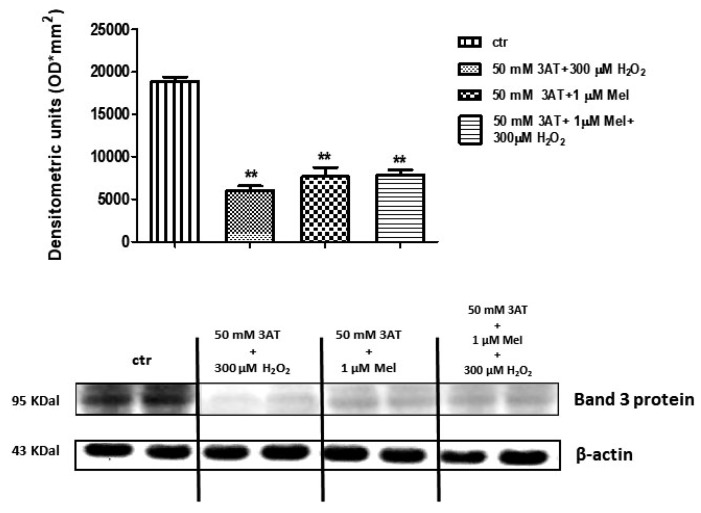
Expression levels of Band 3 protein measured in untreated erythrocytes (ctr), or in 50 mM 3-AT + 300 µM H_2_O_2_- or in 50 mM 3-AT + 1 µM Mel or in 50 mM 3-AT + 1 µM Mel + 300 µM H_2_O_2_-treated erythrocytes, detected by Western blot analysis. ** *p* < 0.01 vs. ctr; as determined by one way ANOVA followed by Bonferroni’s *post hoc* test (N = 5).

Figure 10 shows that Band 3 protein expression levels in either 50 mM 3-AT + 300 µM H_2_O_2_ or 50 mM 3-AT + 1 µM Mel or 50 mM 3-AT + 1 µM Mel + 300 µM H_2_O_2_-treated erythrocytes were significantly lower than those determined in untreated erythrocytes (control) (*p* < 0.001).

**Figure 11:**
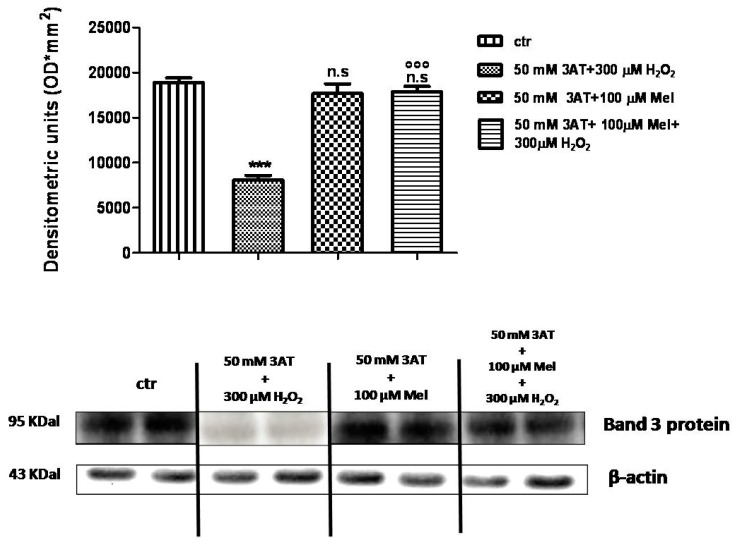
Expression levels of Band 3 protein measured in untreated erythrocytes (ctr), or in 50 mM 3-AT + 300 µM H_2_O_2_- or in 50 mM 3-AT + 100 µM Mel or in 50 mM 3-AT + 100 µM Mel + 300 µM H_2_O_2_-treated erythrocytes, detected by Western blot analysis. *** *p* < 0.001 vs. ctr; n.s. not significant vs. ctr; ^°°° ^*p* < 0.001 vs. 50 mM 3-AT + 300 µM H_2_O_2_, as determined by one way ANOVA followed by Bonferroni’s *post hoc* test (N = 5).

As depicted in Figure 11, Band 3 protein expression levels in either 50 mM 3-AT + 100 µM Mel or 50 mM 3-AT+100 µM Mel + 300 µM H_2_O_2_-treated erythrocytes were not significantly different with respect to those determined in untreated erythrocytes (control), while, after treatment with 50 mM 3-AT + 300 µM H_2_O_2_, they were significantly lower than control (*p* < 0.001). Moreover, a significant difference (*p* < 0.001) between expression levels in 50 mM 3-AT + 100 µM Mel + 300 µM H_2_O_2_-treated erythrocytes and those measured in 50 mM 3-AT + 300 µM H_2_O_2_-treated erythrocytes was observed (*p* < 0.001, Figure 11).

## 3. Discussion

High O_2_ tension in arterial blood contributes to continuous oxidant species production in erythrocytes, including superoxide (O2•), hydroxyl (HO•) and H_2_O_2_ [24], with detrimental effects on cells and tissues when formed in excess [25]. Oxidative damage has been shown to reduce rheological properties, survival of circulating erythrocytes, pathophysiology of abnormal erythrocytes [26] and to affect erythrocytes shape which critically correlates with Band 3 protein function [4,22,27]. Hence, due to their sensitivity to oxidation, erythrocytes are a unique cell model [20] to investigate the impact of oxidative stress and to possibly assess the efficacy of antioxidant therapies. In this regard, Band 3 protein function alterations have been correlated to both in vitro oxidative conditions and oxidative stress-related diseases, i.e., systemic sclerodermia, diabetes and canine leishmaniasis [8,9,28].

As antioxidant supplements may support the endogenous antioxidant system in preserving cell homeostasis under high oxidative conditions [29], the aim of the present investigation was to evaluate the effect of Mel against H_2_O_2_-induced oxidative damage on anion exchange capability through Band 3 protein in human erythrocytes [4,5,22]. To accomplish this aim, pharmacological doses of Mel have been employed (µM) in line with what already reported in both in vitro and in vivo investigations [15]. In particular, the choice of µM Mel doses is supported by da Silva and co-authors [19], who recently described a beneficial effect of 100 µM Mel on erythrocytes exposed to oxidative stress, as well as by Venegas and co-authors [30] reporting about the therapeutical effect of Mel administered in rats at a µM dose.

It is known that Mel, whose role has been widely investigated [12,13,14,15,16,17,21,31], exerts both pro-oxidant and antioxidant effects, depending on concentration, time of treatment and cell target [15]. In particular, the evidence of a pro-oxidant action of Mel mediated by ROS production has been obtained on tumoral cells exposed to pharmacological concentrations of Mel (µM to mM range) [15,17,18,21,31,32,33]. On the other hand, utility and efficacy of Mel in preventing MDA (malondialdehyde) production due to lipid peroxidation in diseases or in experimental models have also been documented [34,35].

As the beneficial effect of Mel in preventing oxidative stress associated to lipid peroxidation has been already demonstrated [34,35], the present study aims to know more about the antioxidant action of Mel against oxidative stress not associated to MDA production by an in vitro model performed on human erythrocytes exposed to 300 µM H_2_O_2_ [36].

It has been already demonstrated that Mel differentially distributes in various subcellular compartments with cell membrane as a major site of concentration (even 1 M), followed by mitochondria, nucleus, and cytosol [15]. On this basis, our hypothesis is that Mel, accumulated at cell membrane level at not pro-oxidant concentrations, may possibly protect ion transport systems against oxidative stress. To this end, not hemolytic concentrations of H_2_O_2_ (300 µM) reducing anion exchange capability with neither lipid peroxidation, nor Met Hb formation, nor oxidation of membrane –SH groups, have been used to model oxidative damage as previously validated [4,22].

The present findings show that Mel concentrations lower than 100 μM (1 μM) are not useful to prove a possible antioxidant effect on Band 3 protein, as, from one hand, lipid peroxidation and, on the other hand, pro-oxidant action on membrane protein are observed, in line with other investigations [15,21,31]. Both Mel effects, with the first being lipid peroxidation that occurs putatively via ROS production, and the second being pro-oxidant action on membrane proteins, result in cell shape alterations that likely produce a membrane re-arrangement. This arrangement to an extent affects shape, although the rate constant for SO_4_^=^ uptake remains unchanged. Moreover, low Mel concentrations (1 μM) and erythrocytes deformability have been already correlated by Dikmenoglu and co-authors [21]. Similarly to what is shown here, these authors detected significant MDA levels in erythrocytes treated with 1 µM but not by 100 µM Mel. Hence, cell membrane alterations may reasonably depend on this action.

As a second step, 100 µM Mel, pharmacological dose also used in other in vitro studies [19] has been considered. Mel doses higher than 100 µM, though not causing lipid peroxidation, have not been used as out of pharmacological range used in both in vivo and in vitro investigations [15]. Pre-treatment of erythrocytes with 100 µM Mel, not producing lipid peroxidation, impaired the reduction in rate constant for SO_4_^=^ uptake due to 300 µM H_2_O_2_, showing for the first time a correlation between the Mel effect, oxidative stress, and Band 3 protein anion exchange capability. In particular, the evidence that H_2_O_2_-induced oxidative stress on erythrocytes involves neither lipid peroxidation nor oxidation of membrane –SH groups nor MetHb formation [6] suggests that the beneficial effect of Mel in protecting Band 3 protein should not be related to these mechanisms. Therefore, an interaction of Mel with membrane arrangement can be hypothesized and also supported, as stated above, by light microscope analysis showing cell shape amelioration in erythrocytes treated with 100 µM Mel before oxidative stress application. In line with our observations, other studies have reported the ability of Mel to preserve fluidity in biological membranes, counteracting free radicals-induced rigidity and stabilizing membranes against lipid peroxidation [37]. In this regard, pinealectomy, with consequent reduction in endogenous Mel levels, has been reported to induce an increase in membrane rigidity that can be associated to physiological ageing [38].

The evidence that Mel at 100 µM concentration delays membrane protein degradation and precipitation of hemin onto erythrocytes membrane [39] may explain the preventing effect of Mel against the expected reduction in Band 3 protein expression levels due to 300 µM H_2_O_2_ [22,40], thus corroborating the hypothesis that the beneficial effect of Mel is exerted at the membrane level.

As oxidative stress alters Band 3 protein aggregation with hemoglobin and cytoskeletal proteins and/or induces protein degradation reducing Band 3 protein expression levels [22,41], it can be suggested that Mel, at 100 µM concentration, would protect Band 3 protein conformation and membrane arrangement, reflecting on erythrocytes morphology. However, to explain Mel action, the role of endogenous antioxidant system should not be neglected. As a pivotal role of catalase in defending erythrocytes against 300 µM H_2_O_2_-induced damage has been already proven [22], Mel treatment under oxidative stress has been assayed in the absence of catalase activity. In these experimental conditions, Mel was effective in preventing the reduction of rate constant for SO_4_^=^ uptake due to oxidative stress, protecting Band 3 protein expression levels even in the absence of catalase activity, thus confirming its antioxidant action. Such observation is supported by Miller and co-authors [42] describing that Mel restores antioxidant enzyme activity in erythrocytes of multiple sclerosis patients and indicating that erythrocytes respond to oxidant challenge with selective induction of certain antioxidant enzymes. Mel has been also reported to alter the activity of glutathione reductase and glucose 6-phosphate dehydrogenase [36,43], with enzymatic activity being increased at 0.02 mM and being decreased at 0.08 mM Mel concentration. With regard to catalase enzyme, Emamgholipour and collaborators [44] have demonstrated that the exposure to H_2_O_2_ and Mel caused catalase activity and an expression levels increase in peripheral blood mononuclear cells. The antioxidant effect of Mel here showed is exerted at concentrations lower than those reported by Dikmenoglu and co-authors [21] using Mel concentrations comprised between 100 and 1000 μM to prevent MDA formation in erythrocytes exposed to high-concentrated H_2_O_2_.

In conclusion, these results propose a suitable in vitro model to add more knowledge about the antioxidant power of Mel, namely in case of oxidative stress not associated to lipid peroxidation and confirm that erythrocytes homeostasis can be protected by Mel against oxidative stress, when used at a specified range of pharmacological concentrations.

Moreover, a dual effect of Mel on erythrocytes, when used at pharmacological doses, can be suggested: low doses elicit a pro-oxidant action, while high doses exert a beneficial effect. Such effects have been for the first time detected by monitoring the homeostasis of erythrocytes, whereas the vast majority of investigations focused on Mel antioxidant mechanism of action on nucleated cells, with specific regard to nucleus and mitochondria [15].

Finally, as most of the in vivo studies demonstrate antioxidant actions of Mel at pharmacological doses, Mel may be suggested as a supplemental therapeutic tool in patients with a deficiency of antioxidant enzymes in red blood cells or in oxidative stress-related diseases associated to Band 3 protein alterations.

## 4. Materials and Methods

### 4.1. Solutions and Chemicals

All chemicals were purchased from Sigma (Milan, Italy). H_2_O_2_ was freshly prepared and diluted from 30% w/w.3-amino-1,2,4-triazole (3-AT), specific inhibitor of catalase [45], was dissolved in distilled water and diluted from 3 M stock solution through a series of dilution (10 and 100 mM). DIDS (4,4′-diisothiocyanato-stilbene-2,2′-disulfonate) was prepared in DMSO and diluted from 10 mM stock solution. Melatonin, kindly provided by Prof. S. Cuzzocrea (University of Messina, Messina, Italy), was dissolved in 0.5% *v*/*v* ethanol to prepare 100 mM stock solution. Solvents (ethanol and DMSO) were preventively tested upon erythrocytes at their final concentrations to exclude any damage.

### 4.2. Erythrocytes Preparation

Human blood was obtained from at least 20 healthy volunteers upon informed consent. Blood was collected in heparinized tubes, washed with an isotonic solution (composition in mM: 145 NaCl, 5 glucose, 20 HEPES (4-(2-hydroxyethyl)-1 piperazineethanesulfonic acid), pH 7.4, osmotic pressure 300 mOsm) and centrifuged thrice (ThermoScientific, 1200 g, 5 min) to remove plasma and buffy coat. Erythrocytes were then suspended to 3% hematocrit in isotonic solution, with or without Mel, and addressed to MDA levels determination.

### 4.3. Lipid Peroxidation

To prove a possible lipid peroxidative effect of Mel, TBARS (thiobarbituric acid reactive species) levels, resulting from reaction between TBA (thiobarbituric acid) and MDA (malondialdehyde), the end product of the lipid peroxidation, were measured as described by Almroth and collaborators [46] with few modifications. The assay has been performed on erythrocytes washed, suspended at 3% hematocrit and incubated for 1 h with either 1 µM or 10 µM or 50 µM or 100 µM or 250 µM or 500 µM Mel at 37 °C, in the dark and with gentle shaking. The reaction between MDA and TBA produces TBARS, which is colorimetrically detectable at a 532 nm wavelength. After incubation with Mel at different concentrations, erythrocytes were centrifuged (ThermoScientific, 1000 g, 5 min), suspended to 10% hematocrit in 1 mL distilled water to induce hemolysis, and frozen overnight at −20 °C. After thawing, sample aliquots (200 µL) were treated with 500 μL TBA (1% *v*/*v* dissolved in 1 N HCl) and incubated at 95 °C for 1 h. Samples were then cooled on ice, centrifuged (Eppendorf, 13,000 g, 15 min, 4 °C), and the supernatant spectrophotometrically read at a 532 nm wavelength (Beckman DU 640).

### 4.4. SO_4_^=^ Uptake Measurement

After TBARS levels determination, 1 μM and 100 μM Mel doses have been used to carry out the following experiments.

#### 4.4.1. Control Conditions

A turbidimetric method [3] was used to measure SO_4_^=^ uptake through Band 3 protein. After 1 h incubation at 37 °C with or without (control) 100 μM Mel, erythrocytes (3% hematocrit in isotonic solution) were suspended to the same hematocrit in 35 mL isotonic SO_4_^=^-containing solution, henceforth named SO_4_^=^ medium (composition in mM: 118 Na_2_SO_4_, 10 HEPES, 5 glucose, pH 7.4, osmotic pressure 300 mOsm). At specified time intervals (5-10-15-30-45-60-90-120 min), 5 mL samples of erythrocytes suspension were put in a test tube containing 10 μM DIDS, which is a specific and irreversible inhibitor of Band 3 protein [47], and then kept on ice. After the last sample withdrawal, erythrocytes were washed thrice in cold isotonic solution (ThermoScientific, 4 °C, 1000 g, 5 min) to remove SO_4_^=^ from the external medium and hemolyzed in 1 mL distilled water, while proteins were hydrolysed by 4% *v*/*v* perchloric acid. Cell membranes were discarded by centrifugation (4 °C, 2500 g, 10 min) and the SO_4_^=^-containing supernatant used for turbidimetric analysis. SO_4_^=^ was precipitated by adding 500 μL supernatant from each sample to 1 mL glycerol and distilled water solution (1:1), 1 mL 4 M NaCl plus HCl (hydrochloric acid 37%) solution (12:1) and 500 μL 1.24 M BaCl_2_•2H_2_O. SO_4_^=^ was spectrophotometrically measured at 425 nm wavelength and the absorption converted to [SO_4_^=^] L cells 10^−2^ using a calibrated standard curve obtained by precipitating known SO_4_^=^ concentrations.

The rate constant in min^−1^ was then calculated by the following equation: C_t_ = C_∞_ (1 − e^-rt^) + C_0_, where C_t_, C_∞_ and C_0_ represent the intracellular SO_4_^=^ concentrations measured at time t, 0 and ∞ respectively; *e* indicates the Neper number (2.7182818); *r* is the rate constant describing the process velocity and *t* is time fixed for each sample withdrawal (5-10-15-30-45-60-90-120 min). Rate constant (min^−1^) reported in Table 1 *per* each experimental condition represents the time needed to reach 63% of total SO_4_^=^ intracellular concentration [3]. The total amount of SO_4_^=^ trapped by erythrocytes after 45 min of incubation in SO_4_^=^ medium (at equilibrium) has been also considered (Table 1). [SO_4_^=^] L cells × 10^−2^ represents SO_4_^=^ concentration internalized by 10 mL erythrocytes suspended at 3% concentration.

To prove that SO_4_^=^ was effectively internalized through Band 3 protein, erythrocytes were suspended in SO_4_^=^ medium (3% hematocrit) containing 10 µM DIDS. Successively, 5 mL samples were withdrawn at fixed time intervals (5-10-15-30-45-60-90-120 min) and handled as described for control conditions.

#### 4.4.2. Exposure to H_2_O_2_

To assay the effect of H_2_O_2_, erythrocytes, after 1 h incubation with or without Mel (1 or 100 μM), were exposed to 300 μM H_2_O_2_ for 30 min at 25 °C. Samples were then centrifuged to remove supernatant and erythrocytes re-suspended to 3% hematocrit in SO_4_^=^ medium. SO_4_^=^ uptake was measured as described for control conditions. Results were compared with those from tests performed with H_2_O_2_ in the absence of Mel.

### 4.5. Light microscope Observations

Erythrocytes, treated or not with either Mel alone (1 and 100 μM) or with 100 μM Mel ± 300 μM H_2_O_2,_ were observed under a light microscope (Leica, 400×), at 90 min of SO_4_^=^ medium incubation.

### 4.6. Erythrocytes Membranes Preparation and SDS-PAGE

Erythrocyte membranes were prepared as previously described [48], with slight modifications. Briefly, after treatment with 300 μM H_2_O_2_ with or without pre-exposure to Mel (1 or 100 μM), packed erythrocytes were diluted into 1.5 mL of cold hemolysis solution (2.5 mM NaH_2_PO_4_) containing a protease and a phosphatase inhibitor cocktail (1 mM PMSF, 1 mM NaF, 1 mM sodium orthovanadate) and then repeatedly centrifuged (Eppendorf, 4 °C, 18,000 g, 10 min) to discard hemoglobin. Membrane were then solubilized by 1% (*v*/*v*) SDS (Sodium Dodecyl Sulfate), and incubated on ice for 20 min. Solubilized membrane proteins, contained in the supernatant, were used for protein content quantification [49] and frozen at −80 °C until use. Membranes obtained from each experimental condition, once thawed, were solubilized (1:1 volume ratio) in Laemmli Buffer [50], heated for 5 min at 95 °C and 2 μg proteins loaded for anti-Band 3 protein. Samples were then separated on 12% polyacrylamide gel and transferred to a PVDF (polyvinylidenefluoride) membrane.

### 4.7. Western Blot Analysis

Membranes were incubated at 4 °C overnight with monoclonal anti-Band 3 protein (1:100,000; Santa Cruz Biotechnology, Dallas, TX, US, produced in mouse), 5% (*w*/*v*) non-fat dried milk and 0.1% Tween-20 and successively incubated with peroxidase-conjugated goat anti-mouse IgG secondary antibodies (1:5,000, AffiniPure, Cambridge, UK), for 1 h at room temperature. To verify whether blots contained equal amounts of protein, they were also incubated with monoclonal anti-β-actin antibodies (1:1000 Santa Cruz Biotechnology) produced in mouse. Signals were detected by a chemiluminescence detection system (Super Signal West Pico Chemiluminescent Substrate, Pierce Thermo Scientific, Rockford, IL. USA). Protein bands expression (approximately 95 kDa), imported to analysis software (Image Quant TL, v2003) and standardized to β-actin levels, was determined by densitometry (Bio-Rad ChemiDoc™ XRS+).

### 4.8. Catalase Inhibition

In order to prove the antioxidant effect of Mel when catalase activity was inhibited, tests (measurement of rate constant for SO_4_^=^ uptake, light microscope observations and Western blot analysis) were carried out by exposing erythrocytes (3% hematocrit) to 50 mM 3-aminotriazole (3-AT) for 10 min at 25 °C, before incubation with Mel (1 or 100 μM) ± 300 µM H_2_O_2_.

### 4.9. Experimental Data and Statistics

Data are expressed as arithmetic means ± S.E.M. for statistical analysis. GraphPad Prism software (version 5.00 for Windows; San Diego, CA, USA) was used. Significant differences between means were tested by one-way analysis of variance (ANOVA), followed by Bonferroni’s multiple comparison *post hoc* test. Statistically significant differences were assumed at *p* < 0.05; *N* represents the number of independent experiments.

## Figures and Tables

**Table 1:** Rate constant for SO_4_^=^ uptake and amount of SO_4_^=^ amount internalized by erythrocytes in different experimental conditions.

Rate Constant (min^−1^)		Time (min)	% Decrease	N	SO_4_^=^Amount Internalized at 45 min of SO_4_^=^Medium Incubation [SO_4_^=^] L cells × 10^−2^
Control	0.076 ± 0.001	12	0	10	201.3 ± 21
1 µM Mel	0.080± 0.001 ^n.s.^	12.5	0	8	192.8 ± 23 ^n.s^
100 µM Mel	0.072 ± 0.001 ^n.s.^	13	0	8	229.8 ± 14.5 ^n.s^
300 µM H_2_O_2_	0.046 ± 0.001 ***	21	40	8	142.2 ± 22.2 ***
1 µM Mel + 300 µM H_2_O_2_	0.048± 0.001 ***	20	37	8	142.8 ± 21.2 ***
100 µM Mel + 300 µM H_2_O_2_	0.074 ± 0.001^§§§^	13.5	0	10	176.2 ± 6.3 ^n.s.^
50 mM 3-AT + 300 µM H_2_O_2_	0.018 ± 0.001 ***	55.5	77	9	58 ± 12 ***^,###^
50 mM 3-AT +1 µM Mel + 300 µM H_2_O_2_	0.019± 0.001 ***	52.6	75	8	38.4 ± 11 ***
50 mM 3-AT +100 µM Mel + 300 µM H_2_O_2_	0.077 ± 0.001 ^n.s.^	12	0	8	219 ± 17 ***
10 µM DIDS	0.017 ± 0.001 ***	55	78	5	4.75 ± 9 ***

Data are presented as means ± SEM from separate *N* experiments, where: ***p < 0.001 significantly different vs. control; n. s. not significant vs. control; ^§§§^*p* < 0.001 vs. 300 µM H_2_O_2_ and control; ^###^*p* < 0.001 vs. 50 mM 3-AT +100 µM Mel + 300 µM H_2_O_2_ as determined by one way ANOVA followed by Bonferroni’s Multiple Comparison *post hoc* test.
